# Relationship between TEC jumps and auroral substorm in the high-latitude ionosphere

**DOI:** 10.1038/s41598-020-63422-9

**Published:** 2020-04-14

**Authors:** A. A. Chernyshov, W. J. Miloch, Y. Jin, V. I. Zakharov

**Affiliations:** 1 Space Research Institute of the Russian Academy of Science, Moscow, Russia; 2 West Department of Pushkov Institute of Terrestrial Magnetism, Ionosphere and Radio Wave Propagation, RAS, Kaliningrad, Russia; 3 Department of Physics, University of Oslo, Oslo, Norway; 4 Faculty of Physics, Lomonosov Moscow State University, Moscow, Russia

**Keywords:** Environmental impact, Aurora

## Abstract

The influence of an auroral substorm on the total electron content (TEC) jumps and cycle slips on Global Positioning System (GPS) at high-latitudes is studied. For the first time, optical data from the all-sky imager, as well as interplanetary magnetic field and magnetometer data are used to complete the analysis of the slips occurrence and to monitor the substorm evolution. Two types of slips are considered: (i) instrumental slips including losses in the measured phase of the GPS signal and (ii) sharp TEC variations (TEC jumps) It is demonstrated that the jumps in TEC determined from the GPS signals are mainly related to the auroral particle precipitation that normally occurs during geomagnetic substorms in the polar ionosphere. The GPS frequency ${L}_{2}$ is consistently subject to more slips than frequency ${L}_{1}$ both for quiet and disturbed conditions. The probability of TEC jumps is higher than for cycle slips in phase at frequencies ${L}_{1}$ and ${L}_{2}$. The maximum of TEC jumps is observed during the recovery phase of the auroral substorm. Our findings are based on a data set obtained for a particular event. A generalization of the obtained numerical estimates to other events requires additional research and further analysis.

## Introduction

The use of Global Navigation Satellite Systems (GNSS) is convenient in solving various problems of both applied and scientific nature, and as such constant improvements in the GNSS accuracy and reliability are vital. The quality of the received GNSS signals is related to the state of the propagation medium, which is the earth’s ionosphere. For example, the Global Positioning System (GPS) uses two frequencies: *f*_1_ = 1575.42 MHz and *f*_2_ = 1227.60 MHz, and provides high accuracy measurements primarily of the phase advance that occurs along the line of sight between the ground-based receiver and the transmitters onboard the GPS satellite^1–3^. Such measurements can be done simultaneously by the two frequency receivers at almost every point of the globe at any time.

Electromagnetic waves transmitted by the GNSS satellites have wavelengths of the order of decimeters, travel through the ionosphere, which is the ionized part of the atmosphere, before reaching a ground-based receiver. Free electrons in the ionosphere can significantly impact on the group, phase and amplitude characteristics of such waves. The ionosphere leads to the group delay and phase advance of GNSS signals. These measurements can be used to calculate the total electron content (TEC)^1,4^, which is an integrated electron density along the ray path between the receiver and satellite determined for a tube with a cross section of one square meter. On the other hand, irregularities in the ionospheric plasma density distort electromagnetic waves during trans-ionospheric propagation and can lead to a poor determination of parameters or even to a temporary discontinuation (slip) of the received GNSS signal^5^.

The main sources of ionospheric irregularities at high latitudes are disturbances of the near-Earth space of helio- and geophysical origins. The near-Earth plasma is affected by the solar wind and the interplanetary magnetic field (IMF), and different physical phenomena, such as geomagnetic storms and substorms, can characterize the state of the near-Earth space environment in relation to the solar activity, which is commonly referred to as “space weather”^6–9^. One of the observable effects of dynamical phenomena in the near-Earth space plasmas are scintillations of transionospheric radio signals. Scientific literature includes a number of studies addressing the stability of the GNSS operation and the accuracy of positioning under disturbed ionospheric conditions^5,10–14^. Scattering of the signal on small-scale plasma density inhomogeneities can lead to strong scintillations of the amplitude and phase of the transionospheric radio signals^15,16^. If scintillations are large enough, they can even result in the signal loss^17^.

The high–latitude ionosphere is characterized by intense small-scale irregularities and inhomogeneities in the plasma density^18–20^, which can be related to electric fields and increased precipitation of energetic particles. For example, the largest number of jumps in determining TEC during periods of geomagnetic storms is located close to the auroral oval^14^. Thus, it is important to carry out detailed studies of ionospheric effects on the navigation signal propagation at high–latitudes^11,21,22^.

The TEC jumps obtained from the GPS signal measurements were studied by Zakharov *et al*.^16^ for years 2010–2014 using receivers located in the Arctic, including also the analysis of probabilities of the instrumental phase and pseudorange (measured distance between satellite and receiver) losses and short-term variations in the high rate TEC measurements at different geomagnetic conditions. It was demonstrated that the probability of big jumps in TEC is substantially (100–200 times) higher than the instrumental GPS slips, and it increases during geomagnetic storms and substorms. Comparison of slips in the GPS and GLObal NAvigation Satellite System (GLONASS) navigation signal, as well as jumps in TEC measurements, was presented in^23^, where the analysis was carried out for both high– and mid-latitude stations under different geophysical conditions between November 2014 and July 2015. Under quiet geomagnetic conditions, the TEC jumps density at mid-latitudes is not higher than 12% and is almost independent of the behavior of *K*_*p*_ or *AE* indices. At high latitudes, the TEC jumps density is higher in winter than in summer and reaches up to 50–60%. Also, the TEC jumps density is 2–2.5 times higher at high latitudes than at mid-latitudes. The authors in^24^ addressed the TEC disturbances in the polar ionosphere using GPS, before and during a magnetic storm on April 5, 2010. It was shown that for the substorm onset during both non–storm and storm periods, there is a significant enhancement of TEC scintillations characterized by the time derivative of TEC since the navigation signals experience stronger scintillations upon traveling through an irregular medium such as in the ionosphere at high-latitudes. The GPS signal phase jumps, resulting in TEC jumps (more than 1 TECU/min), occur predominantly inside the auroral oval and in the vicinity of its equatorward boundary.

Consequently, operational slips include two types of events^16,17^ that will be studied here. The first type of events is the “instrumental” slips that include losses of measurements of distance to the satellite and the phase of the navigation signal, which are observed as a short-term absence of these parameters (pseudo-ranges and phases) measured at a given operating frequency. We refer to them as slips in phases L1 and L2. In this work, we analyze the breakdowns in tracking of both operating frequencies and ranges to the GPS satellite estimated by the receiver.

The second type of events is associated with the scientific use of GNSS signals for characterizing the near-Earth space plasma. They are largely related to the study of TEC variations obtained from the received GNSS signals. Consequently, the second type of slips can be seen as sharp TEC variations or jumps that do not have a physically-sound explanation or are possibly related to yet unknown processes or phenomena (we refer to them as TEC jumps). To identify such cases, we introduce a threshold above which the statistics of the indicated jumps are considered. Since the estimates of regular variability in the ionosphere, for example at sunrise or sunset, give values that are usually less than 1 TECU/min, we set a threshold for TEC jumps at this value in the present study. Due to high noise in the measurements of pseudorange, it is of little use to employ the the group measurements of TEC for such an analysis, and thus we use only the phase measurements.

In the present work, we study in detail the effects of a geomagnetic substorm that occurred on December 23, 2014, between 19:00 and 23:59 universal time (UT), on the slips in phases L1 and L2 as well as TEC jumps at high latitudes. To complete the analysis, the optical data from the all-sky imager and magnetometers are used to monitor the evolution of the substorm. We focus on the response of the GPS navigation system to the perturbed ionosphere and study the TEC jumps, calculated from the GPS navigation signals.

## Instrumentation and Research Methodology

In this section, we describe in detail the scientific instruments and their location, as well as research methodology used in this study.

### Instrumentation

For studying the auroral morphology and the evolution of auroral substorm, we use the all-sky imager (ASI) located at Skibotn, theground-based magnetometer at Tromso, and the OMNI IMF data. ASI has been operating in Skibotn since October 2014. It consists of the electron multiplying charge-coupled device with high signal-to-noise ratio and narrow-band optical filters^25^. ASI records images at wavelengths of 630.0 nm and 557.7 nm every 30 and 15 seconds, respectively. The exposure times are 2 seconds for images at 630.0 nm, and 1 second for images at 557.7 nm.

ASI is collocated with the GPS scintillation receiver at Skibotn. The GPS scintillation receiver is the standard GPS Ionospheric Scintillation/total electron content (TEC) Monitor (GISTM), model GSV4004^26^. GISTM computes and records the amplitude and phase scintillation indices as well as TEC from the dual frequency measurements. In this study, we only use the TEC data which has temporal resolution of 1 second. We post-process the data for intervals of 30 seconds.. Both ASI and scintillation receiver are operated by the University of Oslo.

**Figure 1:**
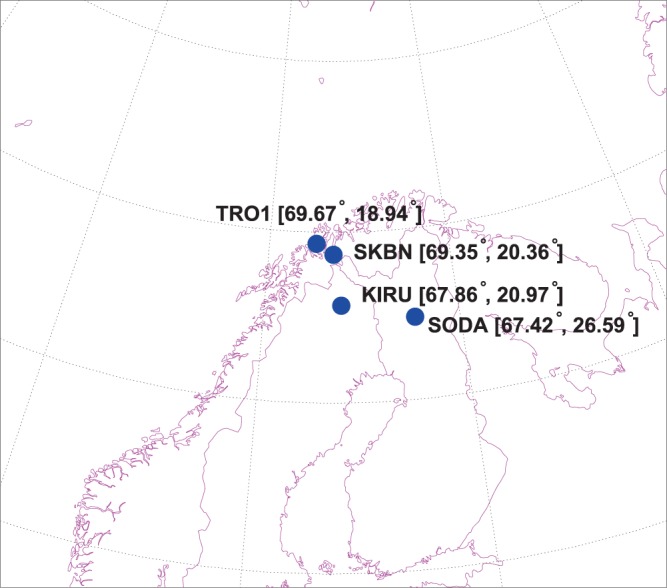
Locations of the GPS receivers used in this study.

Finally, to study the spatial variability of the GPS TEC measurements, we use in total 4 GPS receivers in the Scandinavian sector, in Tromsø (TRO1, 69.663°N, 18.940°E), Skibotn (SKBN, 69.348°N, 20.363°E), Kiruna (KIRU, 67.857°N, 20.968°E) and Sondankyla (SODA, 64.879°N, 21.048°E). For clarity, the locations of all receivers are indicated in Fig. 1. While the data from Skibotn is taken directly from the receiver with the resolution of 1 second, data from other GPS receivers are taken from the International GNSS Service (IGS) in the Receiver Independent Exchange Format (RINEX) format (http://www.igs.org/). The RINEX files provide data with a resolution of 30 seconds. Therefore, to ensure statistical uniformity, we used a standard resolution of 30 seconds in all of our calculations.

### GPS TEC jumps

The ionosphere causes the delay of radio navigation signals traveling from a satellite to a receiving station. The magnitude of this ionospheric delay is proportional to the total electron content, TEC, along with the propagation path, which can be calculated using measurements of the phase of the GPS carrier wave. TEC calculation from the two-frequency phase measurements of the GPS signal^1^ is given by the following formula:

1 $$TEC=\frac{1}{40.308}\cdot \frac{{f}_{1}^{2}\cdot {f}_{2}^{2}}{({f}_{1}^{2}-{f}_{2}^{2})}\cdot [({L}_{1}{\lambda }_{1}-{L}_{2}{\lambda }_{2})+K+nL]$$

where *f*_1_ = 1575.42 MHz and *f*_2_ = 1227.6 MHz are the operating frequencies of GPS, (${L}_{1}{\lambda }_{1}$ -${L}_{2}{\lambda }_{2}$) is the propagation difference between two frequencies due to the dispersive nature of the ionosphere, ${L}_{1}$ and ${L}_{2}$ are the measured phases at GPS frequencies, ${\lambda }_{1}$ and ${\lambda }_{2}$ are the wavelengths, *K* is a constant due to the non-unique phase determination, and *nL* is the error in determining the phase path.

TEC measurements are often used to study the ionospheric response to geophysical events^10,13^, noting that the regular variability of the ionosphere (i.e., quiet–time ionosphere) is typically less than 1 TECU/min^16,23,24^. However, in the ionospheric research, the inverse problem is also encountered, which is the study of the impact of the ionosphere on the properties of trans-ionospheric radio signals. One of the approaches is the probabilistic estimate of the navigation signal phase slips or calculated TEC jumps at main operating frequencies. As a rule, the GPS receiver can measure very accurately a fractional part of the carrier phase received from a satellite, and also accumulate the total number of carrier cycles over time^1^. However, varying electron densities along the path from the satellite to the receiver can cause the GPS signals to undergo rapid fluctuations in phase and amplitude, which can result in a loss of lock on those signals and a delay before signals are restored.

Indeed, the determination of TEC is affected by both additional phase uncertainties in (1) and regular properties of the disturbed ionosphere. Note that several cycle slip correction methods were introduced in previous works^27,28^ but in the present study we do not discuss this issue. Thus, slips including the two types of events, which we discussed in the Introduction section, are studied here.

The cutoff elevation mask to a satellite (elevation angle) also is significant for studying the ionosphere. For quiet conditions, the change in ionospheric delay between the satellite and receiver is mainly due to varying elevation angle of the satellite^1^. The receiver starts to track the satellite signal after the satellite rises above the horizon at a certain angle. Usually, the cut–off elevation mask value is set to 10 or 15 degrees for ionospheric studies. However, using lower degree elevation cut–off angle was also suggested^29^ since the maximum error is always less than 2 TECU and it is typically at the 1 TECU level. The advantage of using lower elevation cutoff angles can be twofold: It would be then easier to separate TEC from the satellite-receiver differential delay^29^ and it would also provide better spatial data coverage^24^. On the other hand, even at elevation angles larger than 30 degrees multipath effects (i.e., due to signal propagation along several paths due to scattering and reflections in a medium with inhomogeneities) can result in phase slips and pseudorange distortions due to the signal interference. Furthermore, the uncertainty of the mapping function is larger at low elevation angles, which should be kept in mind when analyzing the results. In this study we choose the cut-off angle of 10 degrees, to get the best coverage and reduce multipath effects.

A comparison of the obtained results of TEC jumps for different elevation angles in the range (5–20 degrees) shows that the probability of jumps increases more than twofold when the elevation angle decreases from 10 to 5 degree which generally coincides with the results in Zakharov *et al*.^16^. The physical explanation can be the proportional increase in the optical path of the signal in the ionosphere.

In this study, we combine phases at the two operating GPS frequencies ${L}_{1}$ and ${L}_{2}$, and subsequently filter the high–frequency noise. To exclude the uncertainty of the initial phase of registered signals, instead of studying TEC itself, we assess the rate of change of TEC (i.e., the temporal TEC derivative *dTEC/dt*)^1,30^. The threshold value is selected based on the following conditions^6,18^:

- the threshold should be low enough to detect small TEC jumps;
- the threshold should be sufficiently large to avoid false slips, which are due to variations and noises in the TEC assessment.

The threshold of 1 TECU/min satisfies the above requirements^23^. Also, the 1 TECU/min level is chosen as an indicator of activity in the medium, which is not associated with regular changes in ionization, due to for example changes in the position of the Sun. By knowing the number of values of the derivative *dTEC/dt* that exceed the threshold value, the jump probability parameter (probability of TEC jumps or slips of measured phase or distance) can be calculated. The probability is statistically significant for a large number of measurements. It is also important that the highest confidence level was picked as the selection criterion of cycle slips and TEC jumps events^31^. Thus, this study involves 5 hours of combined observations of ${P}_{1}$, ${P}_{2}$, ${L}_{1}$, ${L}_{2}$ (data in the RINEX format), which give more than 2500 measurements of the parameters listed above. In addition, we apply the confidence level of 99%. We calculate distributions ${P}_{T,i,GPS}$ of cycle slips and TEC jumps in time for each of the observed satellite (GPS PRN (pseudorandom noise)):

2 $${P}_{T,i,GPS}=\frac{{N}_{i,GPS}(T)}{{S}_{i,GPS}}\cdot 100 \% $$

where ${N}_{i}(T)$ is the total number of slips of the studied signal *“i”* (here *“i”* means ${L}_{1}$, ${L}_{2}$, TEC, ${P}_{1}$ and ${P}_{2}$) recorded for a given GPS satellite within the observation time *T* and ${S}_{i}$ is the total number of daily observations of signal *i* from a tracked GPS satellite. The time interval *T* is selected based on the appropriateness of dataset for statistical calculations and available computer memory. We use the number of observations per day as a normalization, rather than the one hour period. This choice allows us to avoid overestimation in a nonstationary processes of the jumps probability for the observations in incomplete hours of the satellite visibility during the satellite rising or setting. Any slips or phase jumps in the partial hours at the beginning or end of the observation interval would have more weight than similar phenomena in a full hour-period. In addition, the number of observation points for different navigation satellites varies at different hours, and our normalization is devoid of the indicated complexity.

Finally, we estimate the probability of slips in the parameter *“i”* measured at each receiving station for all visible navigation satellites ${N}_{GPS}$:

3 $${P}_{T,i}=\frac{\mathop{\sum }\limits_{j=1}^{{N}_{GPS}}\,{({N}_{i,GPS}(T))}_{j}}{\mathop{\sum }\limits_{j=1}^{{N}_{GPS}}\,{({S}_{i,GPS})}_{j}}\cdot 100 \% $$

Thus, the experimental data allow us also to make the direct estimates of the cycle slips and TEC jumps manifested at each station for all navigation satellites.

**Figure 2:**
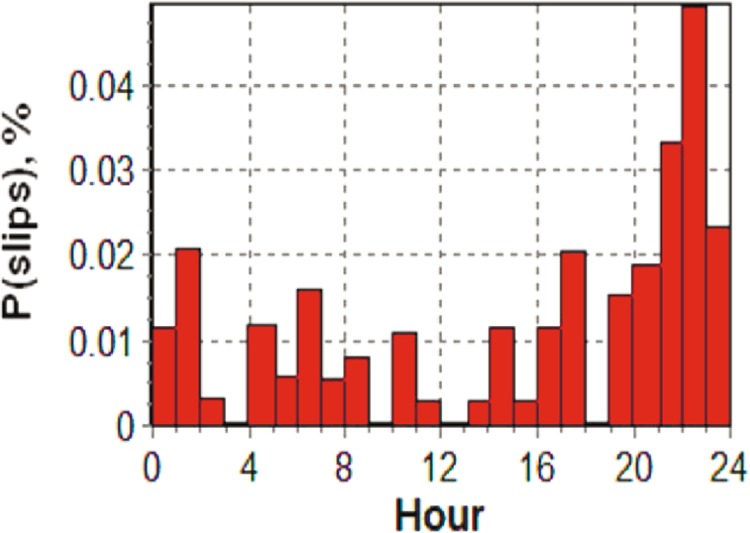
Occurrence of phase slips at frequency ${L}_{1}$ at station SKBN for the whole day.

It should be noted that any slips or phase jumps in the partial hour at the beginning or end of the observation session would have more weight than similar phenomena in a full hour. The fact is that the number of observation points for different navigation satellites varies at different hours, and our normalization is devoid of the indicated complexity and allows reducing the impact of such processes on the overall statistics. An example for the whole day for temporal distribution of phase slips at the frequency ${L}_{1}$ at station SKBN is shown in Fig. 2. Thus, in the results we present only the part that corresponds to the focus of this article, namely, the influence of the geomagnetic substorm on the performance of GPS.

## Results and Data Analysis

**Figure 3:**
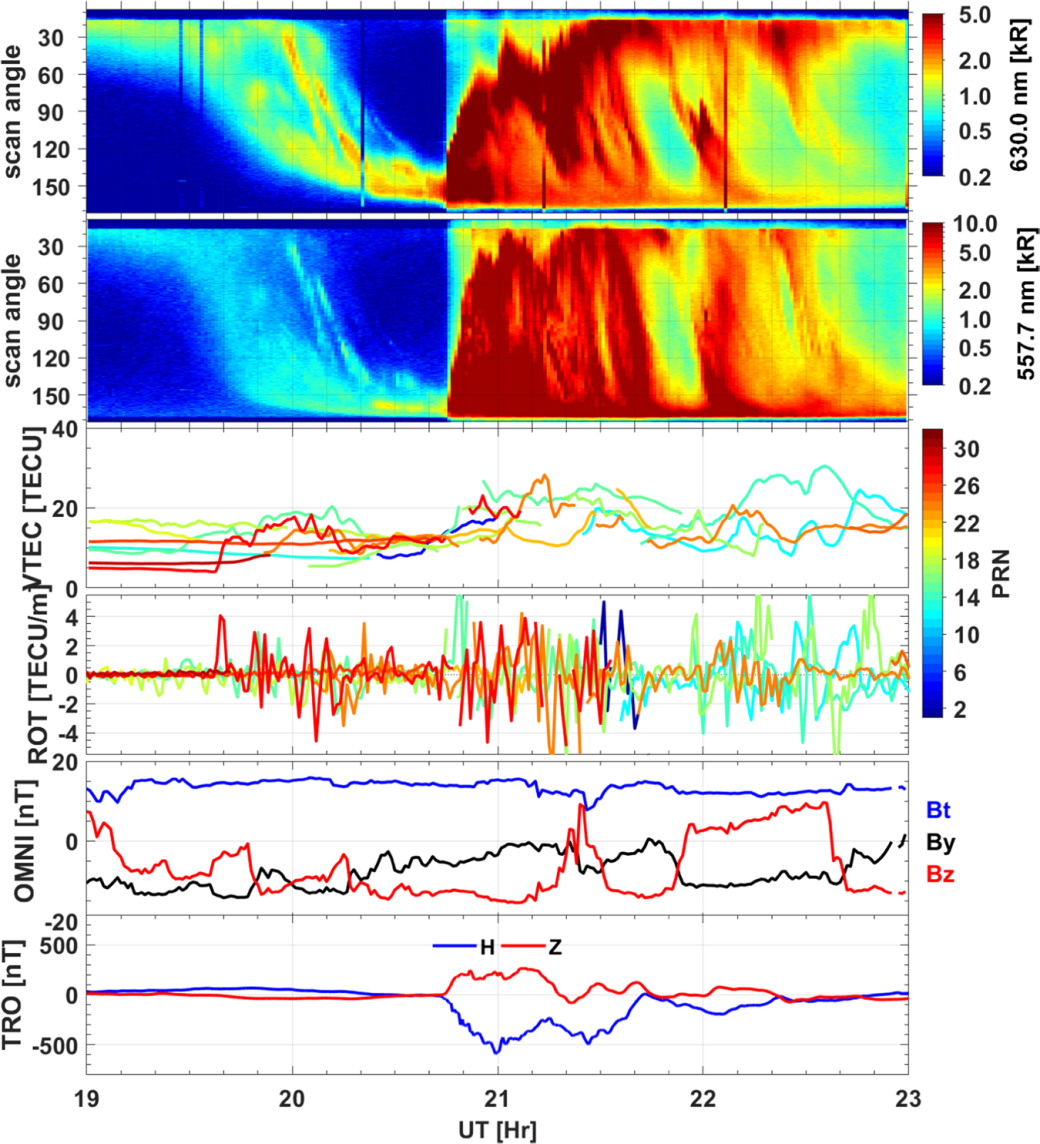
Overview of event on December 23, 2014. From top to bottom: (**a**,**b**) the optical data in a keogram format from the all-sky imager at 630.0 nm and 557.7 nm, respectively (the auroral intensity is indicated by the bars on the right side of each panel), (**с**) GPS TEC from the collocated GPS receiver at Skibotn (the color shows data from different GPS satellites as indicating by the PRN code in the color bar), (**d**) the rate of change of TEC (ROT), (**e**) the OMNI IMF data, and (**f**) the H and Z components of the magnetic field from the ground-based magnetometer in Tromso. Here, nT is nanotesla; nm is nanometer and kR is kilorayleigh.

Figure 3 gives an overview of the studied event. Panels (a) and (b) show the optical data from ASI at Skibotn at 630 nm and 577,7 nm, respectively, which are presented in the keogram format^32^, i.e., temporal evolution of the emission along the magnetic meridian at elevation angles between 15 degrees north to 165 degrees south. The OMNI IMF data are given in panel (e), with the IMF Bz shown in red and the IMF By in black in the GSM (Geocentric solar magnetospheric) coordinate system. The IMF Bz turned southward from 19 UT and remained negative until 21:20 UT. For this orientation of the IMF, we expect the dayside magnetopause reconnection, and the consequent polar cap expansion. This expansion can be clearly seen in the equatorward motion of the auroral oval after 19:30 UT in panels (a-b). The substorm onset was at around 20:45 UT when the aurora suddenly intensified and expanded poleward, after which the whole sky was covered with the auroral arcs. In addition, one can observe that there are several auroral streamers moving equatorward from the poleward edge of the auroral oval. The corresponding ionospheric currents are reflected in the H and Z components of the magnetic field, which are measured by magnetometers at a nearby station in Tromsø (panel f). The sudden decrease in the H component at around 20:45 UT suggests the onset of the magnetic substorm, which developed rapidly, as the H component rapidly reached −500 nT at 21 UT. At the same time, the Z component of the magnetic field increased, implying that the westward auroral electrojet was mainly equatorward of Tromsø. This is consistent with the auroral measurements with the all-sky imager, which show strong aurora in the southern part of the field-of-view. Panel (c) shows TEC from satellites and panel (d) present the GPS TEC Rate Of change of TEC (ROT) measured at Skibotn station. The GPS TEC are characterized by large-scale fluctuations, which are clearly depicted by the ROT in panel (d). These large-scale TEC fluctuations are likely associated with auroral ionization process^33^.

**Figure 4:**
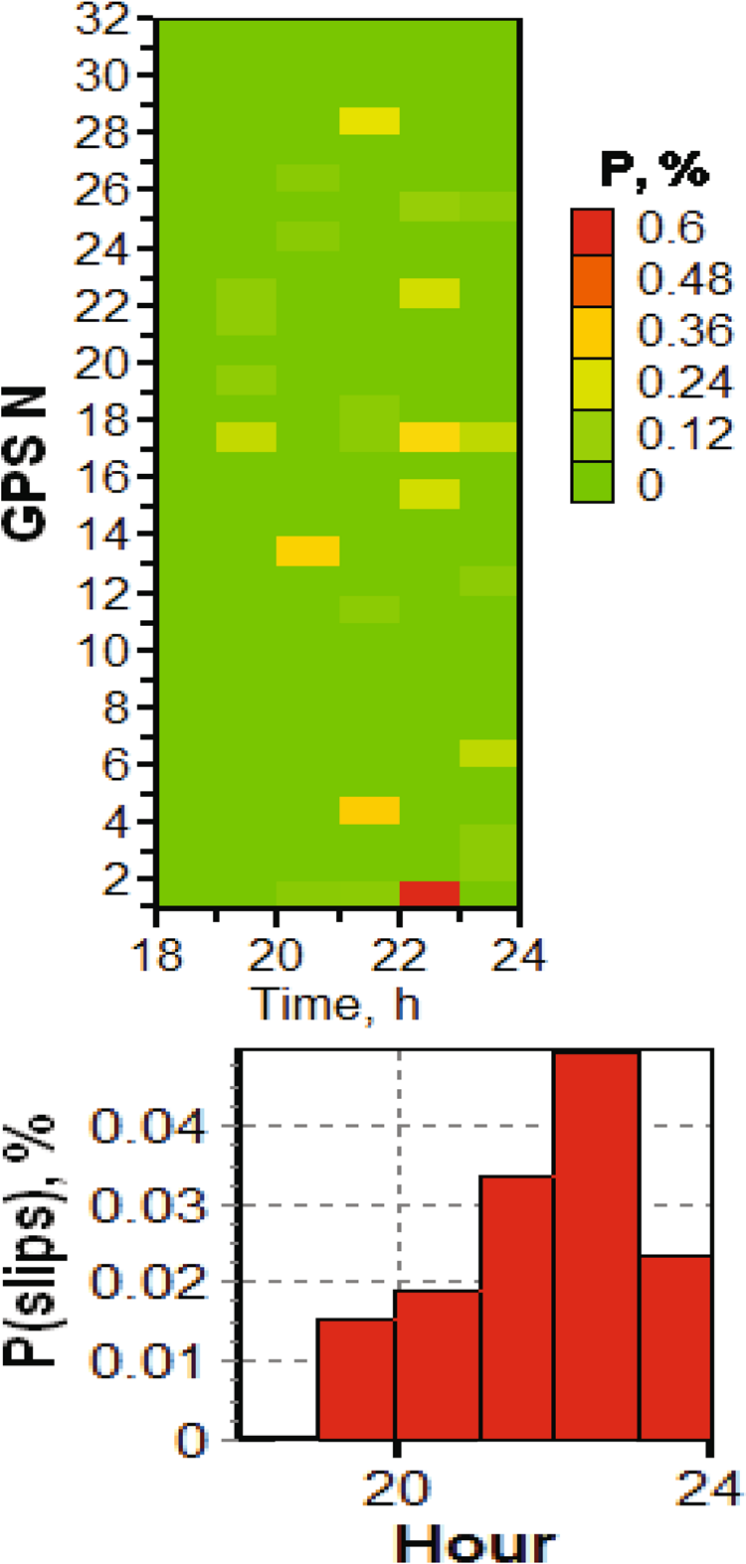
Temporal distribution of phase slips at frequency ${L}_{1}$ (top panel) and averaged probability of phase slips at frequency ${L}_{1}$ (bottom panel) on December 23, 2014 for all visible GPS satellites at station *SKBN*. The elevation angle is above ${10}^{\circ }$.

Having provided a background of geomagnetic conditions, we can proceed with the study of cycle slips in the GPS signals (L1, L2), and estimated vertical TEC by (1) using data from stations TRO1, SKBN, KIRU and SODA (see Fig. 1). In Fig. 4, we present the temporal distribution of the phase cycle slips probability for frequency ${L}_{1}$ measured at Skibotn for selected satellites as well as for all satellites in the field of view, as given by (2) and (3), respectively. The probability of the phase cycle slip at ${L}_{1}$ increased after 19 UT as seen from the upper panel, and the number of slips ${L}_{1}$ increases substantially between 20 to 23 UT, with the peak between 22 and 23 hours. It should be noted that the onset of the magnetic substorm is about 20:45 UT (see Fig. 3), while the auroral oval expansion is observed from 19:30 on December 23, 2014. We observe that the substorm influence on the GPS operations is significant, as the slip probability ${P}_{T,{L}_{1},GPS}$ reaches 0.4% to 0.5% during the recovery phase for selected satellites, which is an increase by 3–5 times as compared to quiet ionospheric conditions. The corresponding average probability for ${P}_{T,{L}_{1}}$ obtained for station *SKBN* is given in Fig. 4, bottom panel. This data relates to all visible GPS satellites at elevation angles $El > {10}^{\circ }$. The average cycle slip probability increases from 0.0175% up to 0.05% during the course of the substorm.

**Figure 5:**
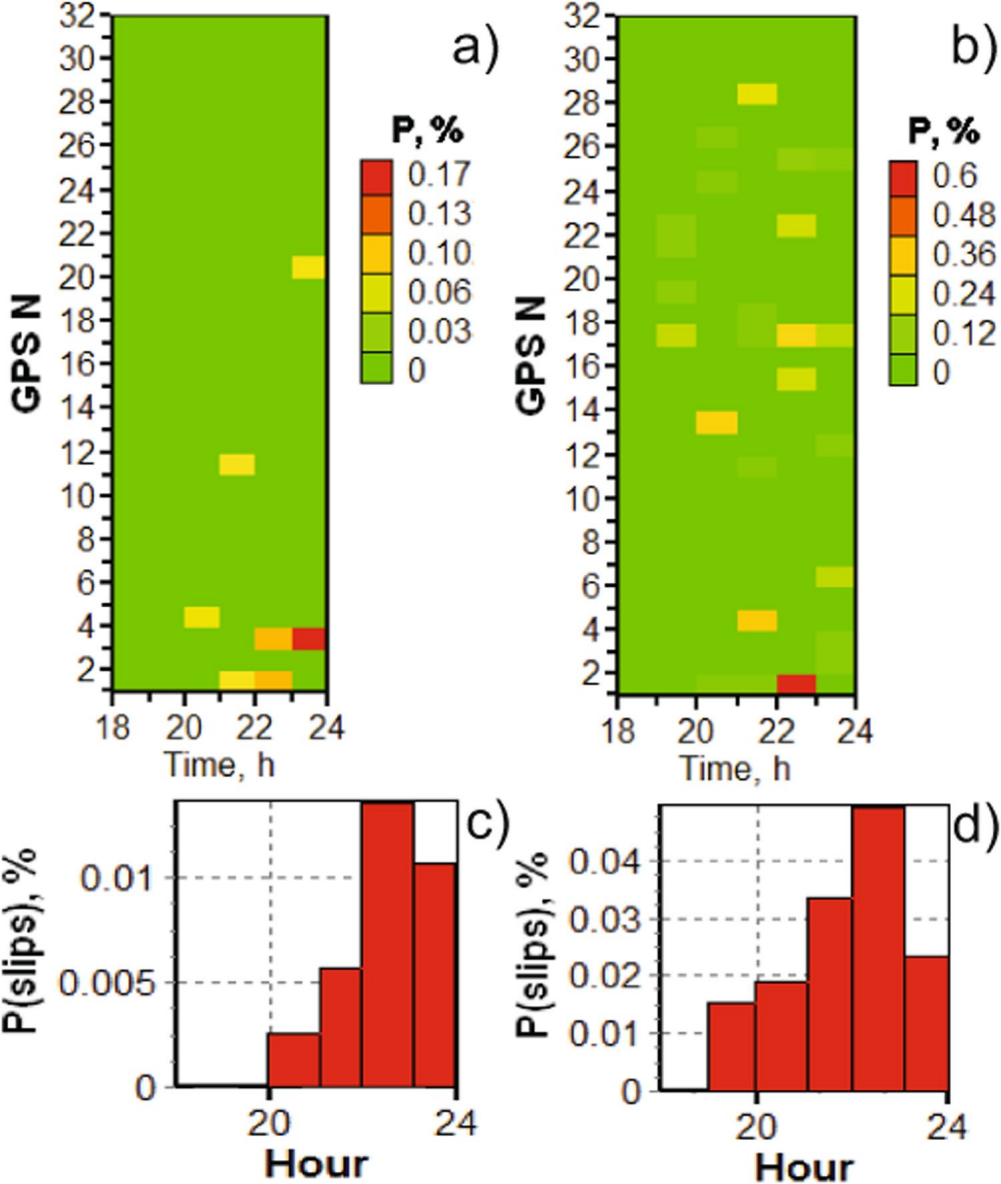
Temporal distribution of phase slips at frequency ${L}_{2}$ at stations *a) SODA* and b) *SKBN*. Averaged probability of phase slips at frequency ${L}_{2}$ at stations c) *SODA* and d) *SKBN* on December 23, 2014 for all visible GPS satellites at stations. The elevation angle is above 10°.

Note that during the studied period, the L1 slips are not recorded at SODA and TRO1 stations, and there are only single events at KIRU, thus we do not present corresponding results for the phase cycle slips at *SODA*, *TRO1*, and *KIRU*. According to the main GPS control document^34^, the GPS system has different powers of routinely radiated signals for the two navigation frequencies, namely ca. 3 dB less for frequency ${L}_{2}$ than for ${L}_{1}$. This, together with the significantly increasing radio power absorption due to the auroral particle precipitation during magnetic substorms, can lead to significant differences in the slip phase probability between the two different operating frequencies. Nevertheless, in Fig. 5, we present data for stations *SODA*, and *SKBN*, and for the phase slips on the frequency ${L}_{2}$ calculated with (2), for the same period of time.

As it is shown in Fig. 5, the probability of the phase cycle slip ${L}_{2}$ increased after 19 UT at *SKBN* and after 20 UT at *SODA*. The maximum number of slips at ${L}_{2}$ is between 21 and 23 UT at *SKBN* and a bit later, between 22 and 23:59 UT at *SODA*. The maximum values are reached before 23 UT for *SKBN* station and midnight UT on for *SODA* station (see Fig. 5a,b). As a response to the substorm onset the cycle slip probability ${P}_{T,{L}_{2},GPS}$ increases from 0.1% to 0.4%. In the recovery/growth phases, the slip probability reaches up to 0.5% (which is an increment by a factor of 5) at *SKBN*. The slip probability ${P}_{T,{L}_{2},GPS}$ at *SODA*, is increased from small values to 0.15%, resulting in an increase in the slip rates by almost factor of 10. The averaged probability of phase slips at frequency ${L}_{2}$ at these stations for all visible GPS satellites and for the elevation angles *El* > 10° on December 23, 2014 gives an assessment of the slip probability ${P}_{T,{L}_{2}}$, which increases from 0.002% to 0.015% at *SODA* and from 0.015% to 0.05% at *SKBN*. In the recovery period, the slip probabilities are respectively 0.014% to 0.05%, as indicated in Fig. 5c,d). It is interesting that the substorm leads to increase in cycle slips ${L}_{2}$ at stations *SODA* and *SKBN* with a delay of several hours (see Fig. 1 for station locations), which is probably associated with the specific features of plasma turbulence originating from geomagnetic disturbances.

**Figure 6:**
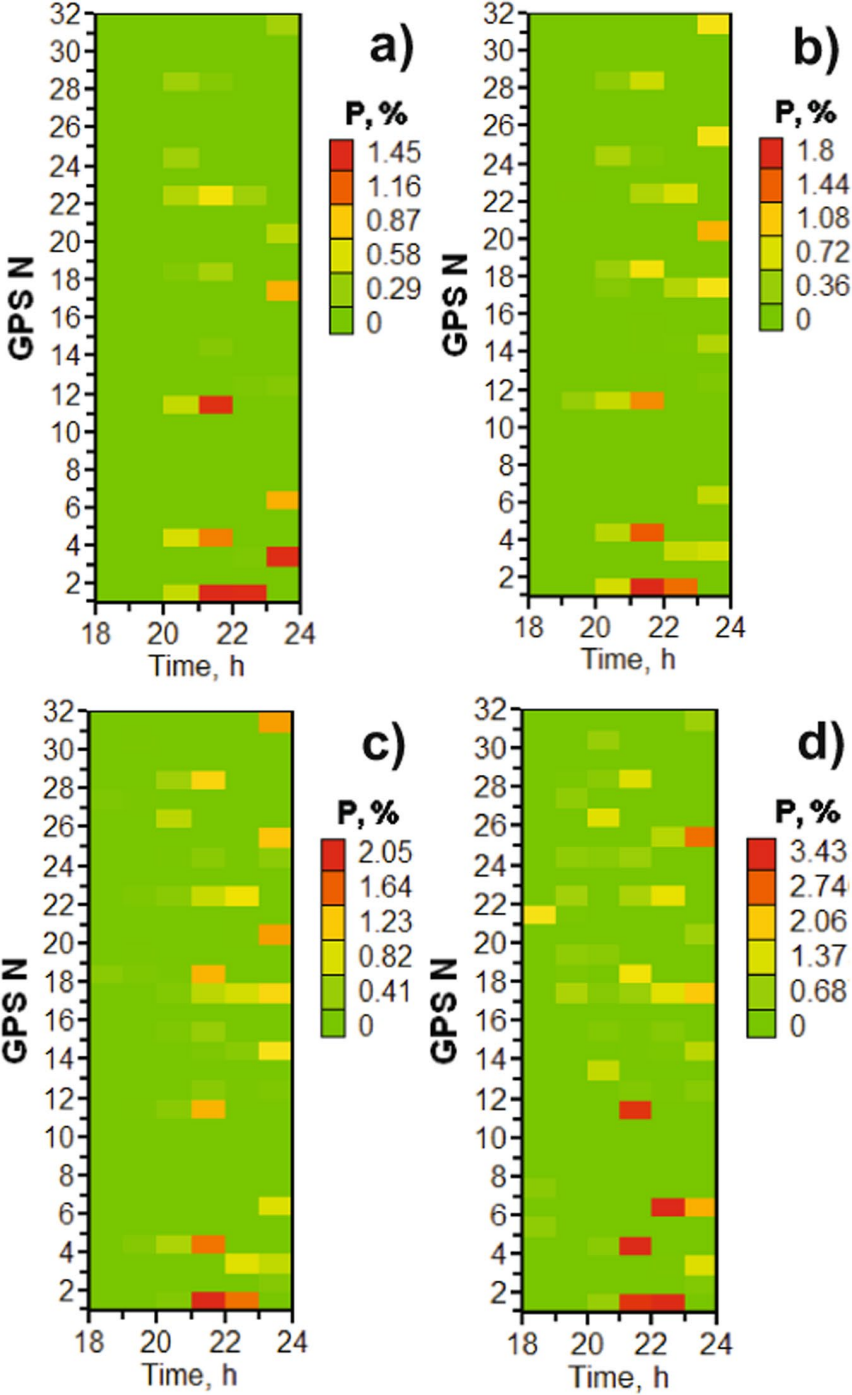
Temporal distribution of TEC jumps on December 23, 2014 for all visible GPS satellites at stations: (**a**) *SODA*; (**b**) *KIRU*; (**c**) *TRO1* and (**d**) *SKBN*.

**Figure 7:**
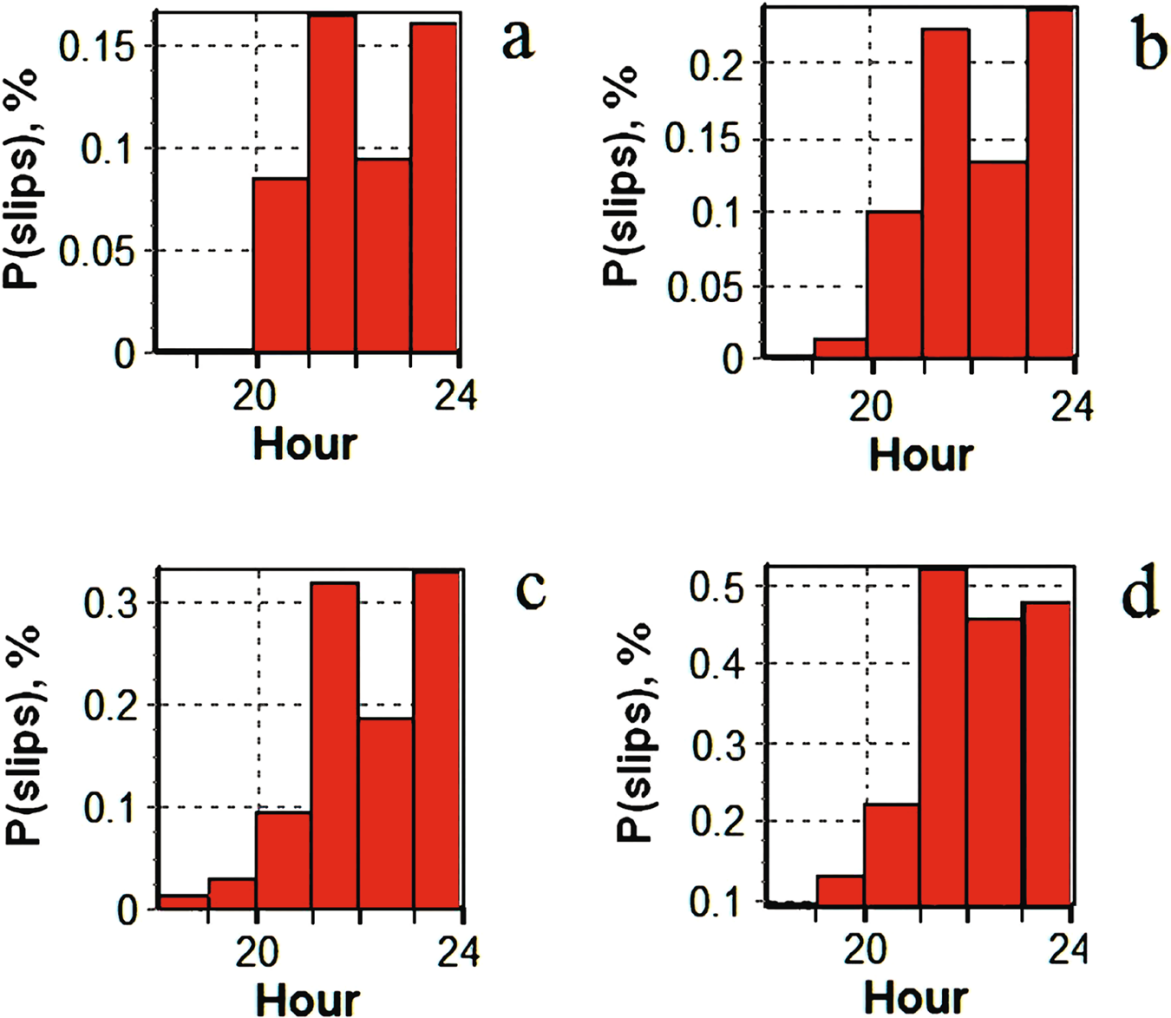
Averaged probability of TEC jumps at stations (**a**) *SODA*; (**b**) *KIRU*; (**c**) *TRO1* and (**d**) *SKBN* for all visible GPS satellites on December 23, 2014.

Figure 6 presents data for the jumps in the total electron content (TEC) from the four stations. It is clear that TEC jumps probability increases with the substorm onset (about 20 UT) and peaks during the recovery and growth phases. Thus, the maximum values for stations *SODA*, *KIRU*, *TRO1*, and *SKBN* are respectively 1.4%, 2%, 2%, and 4%. As a criterion of jumps, we take a level of TEC change of more than 1 TECU/min, and present the example data in Fig. 7, where it is clear that the TEC jumps increase 5–10 times during the substorm as compared to the quiet period. Note that TEC jump means in general an addition turbulization in the electron concentration along the path of the GNSS signal.

It should be mentioned that our study indicates that the probability of TEC jumps is 10 times higher than the probability of cycle slips in phase at ${L}_{2}$ and more than 3 times higher than at ${L}_{1}$ at station *SKBN*. The probability of TEC jumps is 10 times higher than the probability of slips in phase ${L}_{2}$ at station *SODA*.

A correlation analysis was carried out to clarify the linear relationship between the observed TEC jumps and the intensity of magnetic substorm development. In order to conduct a correlation analysis of magnetic substorm development, the average value of the AE index was considered for each of its hours. Then, we determined the correlation coefficient of the obtained averaged indices AE with the probability distribution of slips shown in Fig. 7. It was found that the correlation coefficient between slips (TEC jumps) and the time dynamics of the *AE* index for all the stations examined was more than 0.6. This result was expected, but, as far as is known, the indicated relationship was not estimated.

**Figure 8:**
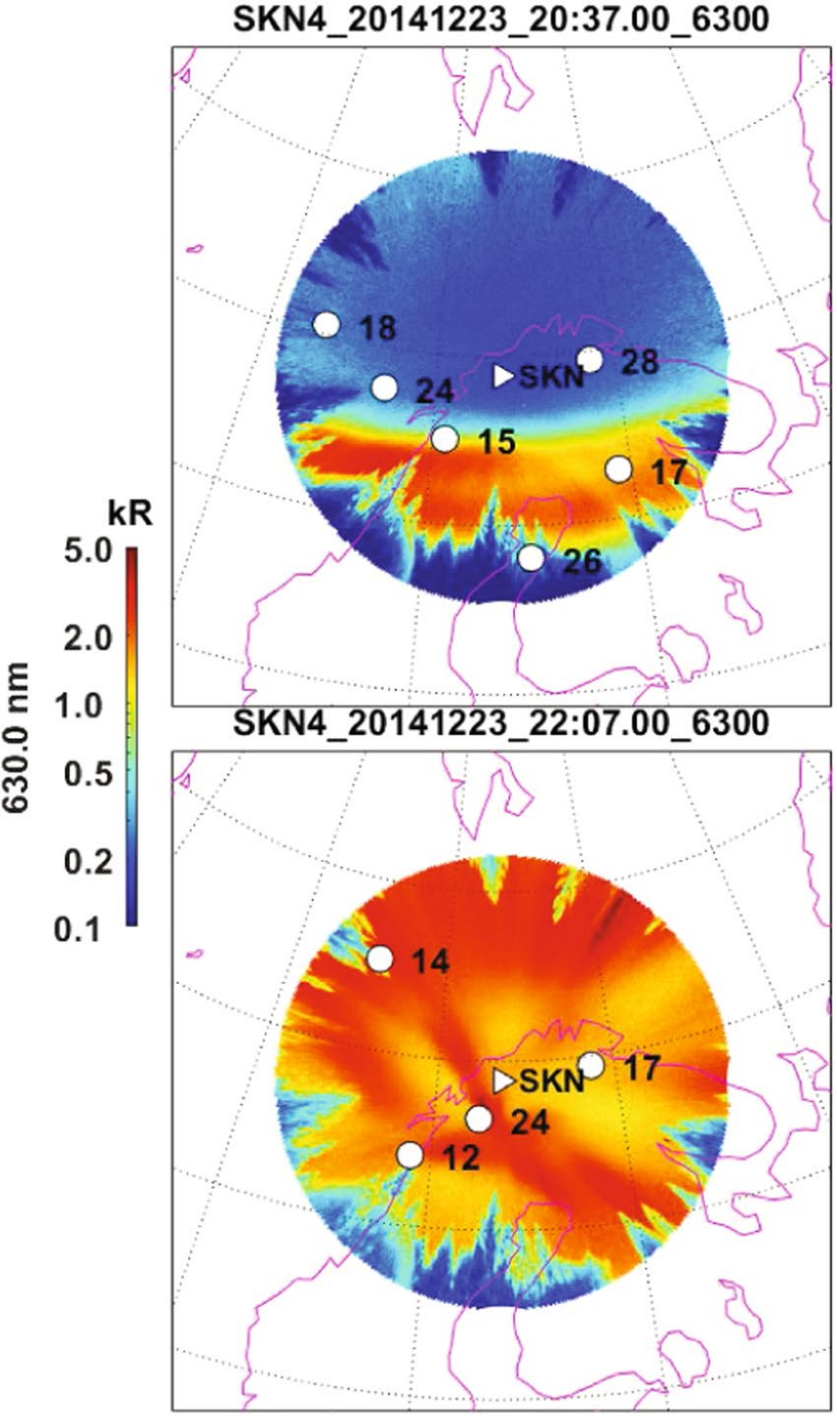
Two selected all-sky images showing auroral emission in 630,0 nm. The ionospheric pierce points of GPS satellites are overlaid. The top panel corresponds to the time just before the substorm onset, while in the bottom panel the substorm auroras covers all the field-of-view at *SKBN*.

For clarity, in Fig. 8, we show the locations of navigation satellites over the studied region and the corresponding optical auroral data. In the upper panel we show data for 20:37 UT, i.e., just before the substorm onset, while in the lower panel for 22:07 UT corresponding to the maximum in the geomagnetic storm when the aurora covered the whole field-of-view at *SKBN*. In the upper panel, a part of the auroral oval is clearly visible. This relates a high number of observed TEC jumps for selected satellites to the auroral phenomena (Fig. 6). It is noteworthy that the slips increase with the transition of the subionospheric point to the main ionospheric trough. The subionospheric points for satellites 15 and 26 are at 20:37 at the borders of sharp changes in glow.

## Discussion

Intense geomagnetic storms and substorms can have a significant influence on the state of the ionosphere and thereby systems relying on the GNSS satellites (GPS, GLONASS, Galileo, BeiDou). Large and fast changes in the plasma density in the polar ionosphere during geomagnetic substorms can alter the propagation velocity of radio signals, introducing the delay in propagation, and lead to variations of phase and amplitude of the GNSS radio signals. These variations are referred to as ionospheric scintillations that can be a source of cycle slips and losses of the carrier lock. The short-term absence of these parameters (pseudo-ranges and phases) measured at a given operating frequency are referred in this work as L1 and L2 (instrumental) phase slips. The scintillations are mainly due to irregularities with sizes of the order of the first Fresnel zone^4,15^. At GPS frequencies of 1.227 and 1.575 GHz, this corresponds to horizontal scales of 150–300 meters in the ionospheric E- and F- layers. Finally, the TEC slips or TEC failures are concerned with TEC data obtained from receiver measurements can be characterized by sharp variations that are lacking the physically-sound explanation.

The current view is that numerous TEC jumps mainly conform to the boundary of the auroral oval, which is the region with significant ionospheric irregularities. In our work, we complement and expand this view by the fact that the slips in the GPS navigation signals can be related to the auroral particle precipitations in the high-latitude ionosphere (northward of the auroral oval boundary); and for the first time, the GPS TEC jumps are correlated with the optical phenomena of the magnetic substorm. It should be mentioned the rarity of the joint operation of all instruments used to study phenomena during a geomagnetic activity. Note that with the use of optical and magnetometer data it is possible to determine the boundaries of the auroral oval.

The auroral particle precipitation leads to an intensification of optical emissions and therefore a relationship between the TEC scintillations and aurora can be expected. A fast moving auroral arc in the ionosphere can generate a short duration diffractive scintillation^35^ and induce a receiver to lose lock when the radio signal travels through the electron density structure related to the aurora^24^.

The present study focused on the response of the GPS signals to the disturbed conditions in the high-latitude ionosphere, with the focus on the relationship between the occurrence of TEC jumps determined by GPS signal and the evolution of a geomagnetic substorm on December 23, 2014. The numerical values of the derivative *dTEC/dt* reached (2–5) TECU/min during a substorm, and such jumps of TEC values exceed the regular variability of the ionosphere, which is typically less than (0.1–1) TECU/min. The statistical data analysis for selected stations gives the mean value of the TEC jumps probability per satellite. Comparing the results from GPS receivers and the optical data indicates that GNSS responds to the disturbed conditions in the polar ionosphere. The essential reasons of TEC jumps are refraction and diffraction due to small structures in the electron density, and the signal absorption in the polar ionosphere due to precipitations of high–energy auroral particles (several keV and higher). Note that the total number of jumps of TEC starts to grow after the onset of the main phase storms.

Ionospheric scintillations result from the scattering of a signal by the electron density inhomogeneities in the ionosphere and lead to a reduction of the signal-to-noise ratio decreasing, in turn, the positioning accuracy. Such effects are being emphasized during geomagnetic storms. There are several studies showing that, due to the influence of unfavorable geophysical factors, the quality of measurements of radio navigation parameters in the frequency range of ${L}_{2}$ deteriorates. For example, it was established^13,36^ that the carrier phase measurement cycle slips occurred mainly at the auxiliary GPS frequency ${L}_{2}$. Phase slips of the signal occurred more often for stations located at high latitudes than at low latitude stations. It was shown that both, the code ranging and phase measurements during geomagnetic disturbances are subject to errors. These results are generally supported by the results of our work.

Note that current ideas about the causes of degradation of decimeter-scale signals are not sufficient for a detailed analysis of mechanisms responsible for the phase and code measurements in the GPS system. As discovered earlier in the paper by Frernouw *et al*.^37^, a highly positive correlation of the growth in the density of phase failures and intensity of TEC variations during geomagnetic disturbances indicates that the increase in the measurement failures can be related to ionospheric plasma irregularities. At the same time, the length of time intervals increases when the signal level becomes lower than the interference level, which causes the signal tracking to fail^36^. However, for the more powerful signal in the *L*_*1*_ band, the duration of such intervals is an order of magnitude smaller than for the less powerful *L*_*2*_ signal. During periods of geomagnetic disturbances of the near-Earth space plasma, due to the appearance of intense inhomogeneities in plasma density, the effect of interference fading of the amplitude of the GNSS signals, as well as the scintillation of amplitude and phase, often prevails. The scintillation statistics in the auroral zone showed a strong correlation between the frequency of their appearance and geomagnetic activity.

The frequency of ${L}_{2}$ is characterized by a smaller signal-to-noise ratio, which makes it a much more sensitive indicator of phase failures. This smaller signal-to-noise ratio at ${L}_{2}$ frequency is primarily due to a lower power of the *L*_2_ transmitter compared to the base frequency transmitter *L*_1_ with the C/A code. A similar difference in the effectively emitted ${L}_{1}$ and ${L}_{2}$ signal powers is typical also for the GLONASS system^10^. Phase failures at ${L}_{2}$ can also be caused by a lower signal-to-noise ratio when commercial receivers are used. These receivers do not have access to the military *Y*-code and are forced to use the codeless or semi-code modes.

### Concluding remarks

In this study, the influence of the magnetic substorm on the TEC jumps and GPS cycle slips in the polar region was investigated together with optical data from the all-sky imager, which was used for a more detailed analysis of the ionospheric conditions. After studying the relationship between cycle slips occurrence in the main parameters of navigation signals, TEC jumps and the optical auroral emissions due to particle precipitation, the main conclusions are:

- TEC jumps and cycle slips in navigation signals can be related to auroral particle precipitations in the high-latitude ionosphere;
- For the first time, we directly relate the GPS TEC jumps to the optical data for auroral emissions in the polar ionosphere during a geomagnetic substorm;
- Frequency ${L}_{2}$ has more cycle slips than ${L}_{1}$ both in quiet conditions and during geomagnetic activity in the polar regions which coincides with the results of other works as well as the localization of “slip spots” in space;
- The TEC jumps probability is several times higher than the probability of cycle slips in phase at the frequencies ${L}_{1}$ and ${L}_{2}$;
- The auroral substorm leads to a growth in cycle slips ${L}_{2}$ at the stations in Sondankyla and Skibotn with a delay of several hours, which may be associated with specific features of plasma turbulence originating from magnetic disturbances;
- The maximum of GPS TEC jumps occurrence is during the recovery phase of a geomagnetic substorm. This is most likely associated with the evolution of plasma inhomogeneities and instabilities in the high-latitude ionosphere excited during the geomagnetic activity.
- The growth of instrumental cycle slips of the phases of the navigation signals ${L}_{1}$ and ${L}_{2}$ and the TEC jumps calculated on their basis during the recovery of the substorm is shown, that is, the dynamics of the development of the substorm is specified, most likely related to the evolution of a developing and relaxing turbulized ionospheric plasma.

Despite the fact that these conclusions are based on a data set obtained for a particular event and further analysis is recommended to confirm and expand the preliminary findings, we can confidently assert that precipitation of energetic particles in the auroral oval play an important role in the structuring of the high-latitude ionosphere and can impact the propagation of transionospheric radio waves. Therefore, monitoring of such geophysical conditions is of high relevance for the wave propagation and performance of the GNSS systems. Thus, it is important to consolidate effort in improving means and subsystems for controlling the integrity of the GNSS signals, as well as differential correction for the rapid detection and effective compensation for cycle slips and TEC jumps and occasional short-term deterioration of the GNSS signals under unfavorable geophysical conditions.
